# Multifocal Kaposiform Hemangioendothelioma Successfully Treated With Sirolimus Monotherapy

**DOI:** 10.1111/pde.70003

**Published:** 2025-08-17

**Authors:** Matthew J. Mahoney, Erin P. Fritz, Alex C. Hoover, Emily G. Greengard, Michael A. Murati, Bradley J. Segura, Daniel D. Miller, John R. Leister, Sheilagh M. Maguiness

**Affiliations:** ^1^ Department of Dermatology University of Minnesota Minneapolis Minnesota USA; ^2^ Department of Pediatrics, Division of Pediatric Hematology/Oncology University of Minnesota Minneapolis Minnesota USA; ^3^ Department of Pediatrics, Division of Pediatric Blood and Marrow Transplant and Cellular Therapy University of Minnesota Minneapolis Minnesota USA; ^4^ Department of Pediatrics, Division of Pediatric Hematology/Oncology University of North Carolina at Chapel Hill Chapel Hill North Carolina USA; ^5^ Department of Radiology University of Minnesota Minneapolis Minnesota USA; ^6^ Department of Surgery, Division of Pediatric Surgery University of Minnesota Minneapolis Minnesota USA; ^7^ Department of Dermatology, Division of Dermatopathology University of Minnesota Minneapolis Minnesota USA; ^8^ Department of Dermatology, Division of Pediatric Dermatology University of Minnesota Minneapolis Minnesota USA

**Keywords:** Kaposiform hemangioendothelioma, Kasabach–Merritt phenomenon, pediatrics, sirolimus, vascular tissue neoplasms

## Abstract

Kaposiform hemangioendothelioma (KHE) is a rare vascular tumor that typically presents in infancy and may be associated with the Kasabach–Merritt phenomenon (KMP). We present a challenging case of multifocal KHE on the leg of an infant, initially suspected at birth to be a reticulate port wine birthmark. Skin biopsy and imaging supported the rare diagnosis of multifocal KHE. Complicated by KMP, he was started on sirolimus monotherapy with significant improvement in his widespread disease.

## 1 Introduction

Kaposiform hemangioendothelioma (KHE) is a rare, locally aggressive vascular tumor, classified as “borderline malignant” by the International Society for the Study of Vascular Anomalies [1]. Despite being locally infiltrative, it rarely metastasizes [1, 2]. While the etiology of KHE remains unknown, several genes have been associated, including sporadic mutations in *GNAQ*, a gene integral to binding G‐protein coupled receptors in tumor and vascular biology, platelet aggregation, and inflammation [3]. KHE commonly presents as an ill‐defined red‐to‐purple papule or indurated plaque, present at birth in about 50% of cases, with most identified within the first year [4].

KHE carries a high rate of morbidity and mortality due to several serious complications, including Kasabach–Merritt phenomenon (KMP), a consumptive coagulopathy often associated with profound thrombocytopenia and hypofibrinogenemia [5]. This occurs in up to approximately 40% of children affected by KHE and is fatal in approximately 1.3% of cases [6]. KHE can lead to mass effect on nearby structures and may infiltrate soft tissues, organs, or bone [4].

Biopsy is the gold standard for diagnosing KHE; however, this is often challenging in cases with KMP or deeper lesions. The histological hallmark of KHE is infiltrating nodules consisting of spindle endothelial cells, leading to malformed lymphatic channels and slit‐like vascular lumina [7]. Immunochemical staining is typically positive for D2‐40, vascular endothelial markers CD31 and CD34, lymphatic endothelial marker VEGFR‐3, lymphatic endothelial hyaluronan receptor‐1, and Prox‐1, but negative for Glut‐1 and HHV‐8 [7, 8].

In the majority of cases, KHE presents as a solitary, infiltrative plaque or tumor. Multifocal presentation of KHE is extremely rare, with few reported cases in the literature. Of those, the majority were clinically diagnosed without confirmatory immunostaining [9, 10, 11, 12, 13, 14, 15, 16], with one case histologically proven [17].

We present a challenging case of multifocal KHE treated with sirolimus monotherapy with excellent clinical response.

## 2 Case Report

A male neonate, born via uncomplicated vaginal delivery, was noted to have a vascular lesion on his left lateral leg and thigh, consisting of blanching erythematous patches with non‐blanching violaceous centers. This lesion was not identified on prenatal ultrasounds.

At 1 week of age, he was seen by pediatric dermatology in teledermatology (due to the COVID‐19 pandemic). Clinical images demonstrated a violaceous, linear‐appearing patch on the left lower leg with an overlying net‐like, reticulate appearance. Per parental report, the lesion had lightened since birth and had not bled or ulcerated. He was preliminarily diagnosed with a reticulate port‐wine birthmark and instructed to follow up at 6 months of age.

At 2 months of age, the infant presented to the emergency department at an outside hospital with an ecchymosis‐like rash on the right flank, lower abdomen, and scrotum. Due to this presentation, there was initial concern for non‐accidental trauma. Despite clinical stability, laboratory studies revealed thrombocytopenia (17 K/uL, reference range 150–450 mg/dL). Prothrombin time (PT) and international normalized ratio (INR) were elevated at 22 s and 2.2, respectively (reference ranges 11.3–15.0 s and 0.81–1.17). He had marked hypofibrinogenemia (30 mg/dL, reference range 125‐300 mg/dL). Computed tomography (CT) of the abdomen and pelvis revealed retroperitoneal enhancement from the upper abdomen to the pelvis, with additional enhancement through the root of the mesentery. He was given a platelet transfusion and was admitted to the pediatric intensive care unit (PICU). His course was complicated by multiple blood product transfusions due to ongoing retroperitoneal bleeding and persistent hypertension requiring a nitroprusside drip.

**FIGURE 1:**
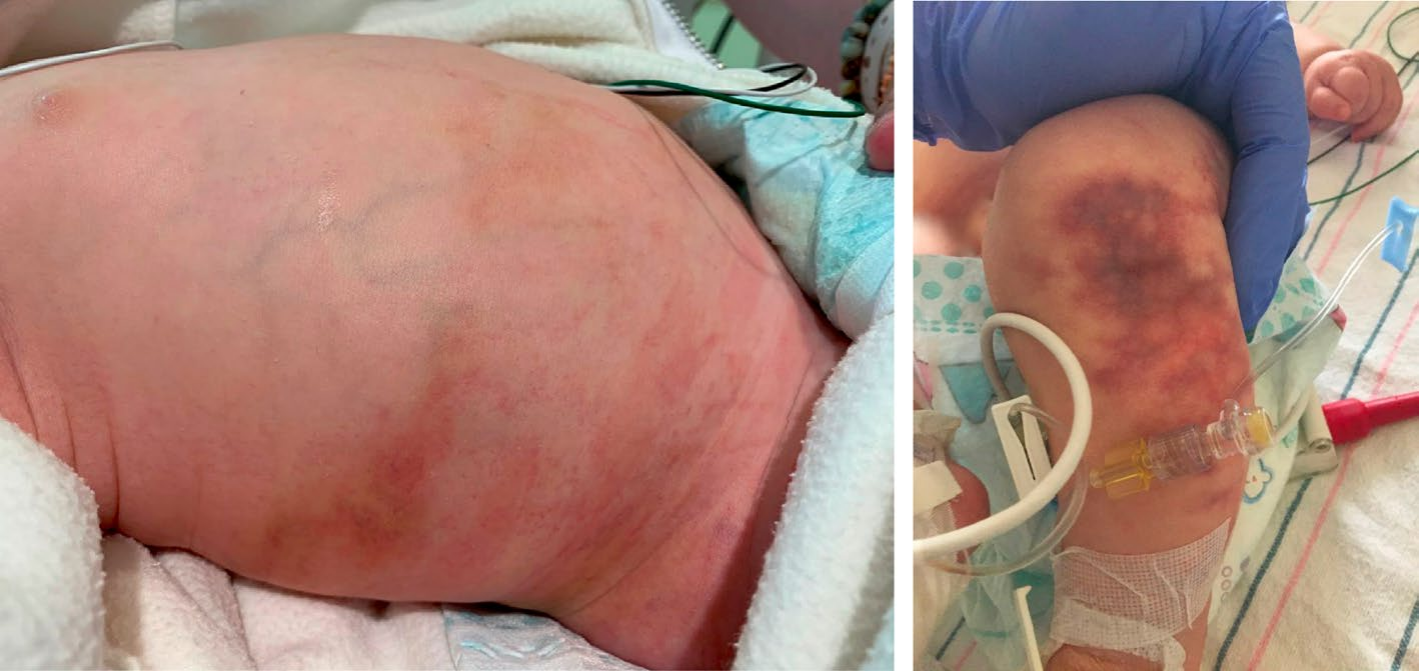
Clinical examination on PICU transfer, demonstrating multiple poorly defined, purple reticulated patches on the right flank (A) and a violaceous, confluent reticulated patch on the left lower extremity (B).

He was subsequently transferred to our tertiary PICU. On initial exam, he was found to have multiple poorly defined, purple reticulated patches without overlying skin changes on the right flank, lumbar back, and abdomen (Figure 1A). There was a firm, violaceous, confluent reticulated plaque on his left lower leg (Figure 1B) without enlargement of his affected leg.

**FIGURE 2:**
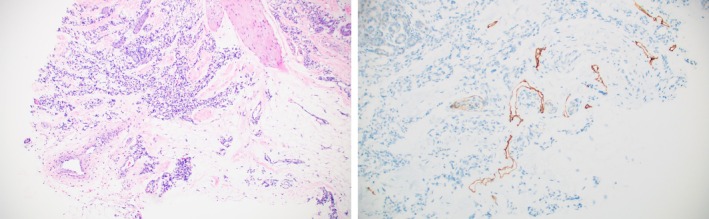
Dermatopathology images from left lower leg biopsy, with (A) showing clusters of small, occasionally slitlike vascular channels infiltrating subcutaneous fat lobules (H&E, ×100), (B) with D2‐40 immunostain demonstrates small slitlike and crescentic lymphatic channels partially encircling tumor lobules.

**FIGURE 3:**
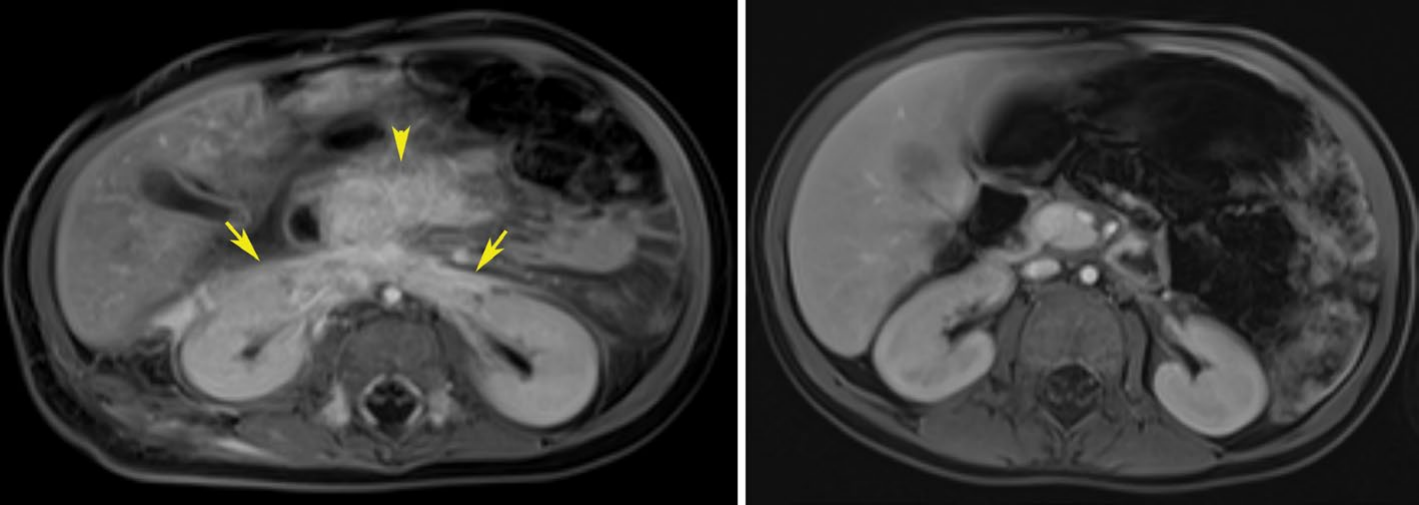
Multiple T1‐weighted axial post contrast images with fat saturation through the upper abdomen. (A) Pre‐therapy MRI at 3 months of age. There is an infiltrative, enhancing mass lesion involving the anterior pararenal space (arrows) with involvement of the pancreatic head and surrounding peripancreatic fat (arrowhead), suspicious for a Kaposiform Hemangioendothelioma (KHE). Not shown is involvement of the mesenteric root and patchy involvement of the right paraspinal musculature. (B) Follow up MRI after 15 months of therapy demonstrates resolution of the infiltrative KHE. The pancreas is now uniform in signal and there is no suspicious enhancement through the mesentery or retroperitoneum.

Magnetic resonance imaging (MRI) revealed an infiltrative and enhancing mass within the retroperitoneum with extension along the mesenteric root and suspected involvement of the pancreatic head (Figure 3A). Several enhancing T2‐weighted hyperintense lesions were noted within the axial and appendicular skeleton. Skin biopsy of the left leg lesion demonstrated a vascular tumor with capillary‐like vascular changes with endothelial cells in fascicles in the dermis and subcutaneous fat with lymphatic channels (Figure 2A). Immunostaining showed a small amount of D2‐40 uptake in lymphatic spaces adjacent to the vascular lobules in the deep dermis and subcutis, not a pattern diagnostic for KHE (Figure 2B). Even so, biopsy findings led to a high suspicion for KHE, and with his ongoing coagulopathy, oral sirolimus monotherapy was started at 0.8 mg/m^2^/dose twice daily, with a goal serum trough level of 8–10 ng/mL.

Throughout his hospitalization, the patient required multiple platelet transfusions given active retroperitoneal bleed, which were cautiously administered given his consumptive coagulopathy. After 2 weeks on sirolimus, the coagulopathy resolved with complete resolution of hypofibrinogenemia and thrombocytopenia. The patient's hypertension was thought to be secondary to the location of the lesion relative to the renal vasculature; although there was no evidence of significant renal artery narrowing on Doppler ultrasound. Follow‐up MRI completed 1 week after starting sirolimus did not demonstrate a change in the size of the lesions.

A follow‐up MRI 3 months after initiating sirolimus showed marked reduction in the retroperitoneal mass with persistence of the osseous lesions. After 9 months on sirolimus, there was continued reduction in the retroperitoneal mass and decreased conspicuity of the bony lesions. MRI, 15 months after treatment initiation, demonstrated resolution of all sites of disease (Figure 3B) and subsequent imaging at 21 and 24 months did not show recurrence. Over 2 years, his cutaneous lesions improved, with only residual pink to violaceous, ill‐defined, reticulate, and stellate patches. Sirolimus was discontinued approximately 2 years after treatment initiation.

Three months after discontinuing sirolimus, follow‐up MRI demonstrated disease recurrence involving the pancreatic uncinate process and multiple lesions in the axial skeleton, similar in distribution to the patient's prior sites of disease. In addition, there had been interval darkening of the reticulate patches on his left lower extremity. Sirolimus was restarted and titrated to an increased goal of 10–15 ng/mL. Despite this, his disease progressed with a new enhancing lesion on his right proximal femur and increased size of numerous enhancing lesions, predominantly in the pelvis. Subsequently, the decision was made to target sirolimus troughs consistently closer to 15 ng/mL.

Nine months after restarting sirolimus, MRI showed resolution of the enhancing lesion on his right femur and stability in all other sites. Improvement continued over subsequent months, and MRIs have shown stability with no evidence of disease progression or recurrence. Given his disease resolution on MRI and development of multiple bacterial infections including pansinusitis and pneumonia, sirolimus was discontinued 26 months after restarting, with a plan for serial MRI every 3 months.

## 3 Discussion

KHE is a locally aggressive endothelial‐derived spindle cell neoplasm that primarily presents in infancy. It remains a rare diagnosis, with a prevalence of 0.91 in 100,000 children [5]. Multifocal disease is exceedingly rare, with few cases reported in the literature [9, 10, 11, 12, 13, 14, 15, 16]. It is challenging to treat, since complete surgical excision is impractical; it is more frequently associated with KMP, and recurrences occur frequently [3, 7]. Our case presents a successful treatment approach with sirolimus alone, avoiding the adverse effects often seen with corticosteroids.

Cases of bone invasion have been reported in the literature, primarily in extracraniofacial sites with associated large soft tissue masses [10]. It has been shown that 26% of pediatric patients with KHE exhibit bone destruction at some point in their disease course, particularly those older than 6 months at the time of diagnosis [18]. Bony invasion is associated with increased morbidity, including decreased range of motion, greater incidence of KMP, and a higher likelihood of tumor rebound [18].

Typically, D2‐40 immunostain in KHE would demonstrate strong immunoreactivity to the endothelial cells that form the characteristic ill‐defined nodules and slit‐like vascular channels [8]. Our case showed an atypical pattern, with only a small amount of uptake in interspersed lymphatic spaces adjacent to the vascular lobules in the deep dermis and subcutis. Ultimately, KHE was supported as the diagnosis despite the non‐diagnostic stain. In our case, metastasis was considered given the multiple lesions in separate locations. However, without histologic features of malignancy on skin biopsy and inconsistent imaging findings, multifocal KHE remained the favored diagnosis.

This case highlights a challenge with teledermatology, the inherent inability to physically assess lesion depth or texture. While teledermatology achieves high diagnostic concordance for common pediatric skin conditions, diagnostic accuracy is significantly decreased for rare or complex presentations, such as KHE [19, 20]. It has been shown that teledermatologists are generally comfortable managing rashes virtually, but have decreased confidence for alopecia, pigmented lesions, and vascular lesions [19]. Cases concerning KHE should be evaluated in‐person if possible, as identifying a deep lesion may lead to earlier biopsy and diagnosis.

In the past, treatment of KHE was limited to steroids, cytotoxic chemotherapeutic agents like vincristine, and interferon‐alpha. In addition to medical management, surgical intervention is occasionally warranted in complex cases [21, 22]. In our case, operative management would have added substantial risk and potential sequelae. In the past decade, studies have increasingly shown the efficacy of sirolimus, a mammalian target of rapamycin (mTOR) inhibitor [2]. Current treatment approaches may utilize sirolimus in conjunction with prednisolone; however, there has been reported success with sirolimus monotherapy [23, 24]. Further research is ongoing to determine treatment length, but studies have shown high‐risk KHE requires at least 2 years of treatment before weaning, a total treatment course of 28 months, and approximately 50% of cases had recurrence with discontinuation [25]. In our case, sirolimus monotherapy led to the complete resolution of KHE over the course of 2 years; however, recurrence was seen upon discontinuation of treatment. Within 9 months of restarting sirolimus, there was evidence of clinical and radiologic response. Over subsequent months, imaging demonstrated improvement and eventual resolution in all sites of disease.

In summary, this case of multifocal KHE and associated KMP included a large cutaneous lesion at birth, bony involvement of the axial and appendicular skeleton, and a retroperitoneal mass. He was treated successfully with sirolimus monotherapy, and his symptoms were entirely resolved at 24 months of age. Multifocal recurrence occurred upon discontinuation of sirolimus; however, it stabilized and improved again after resuming sirolimus monotherapy.

## Conflicts of Interest

The authors declare no conflicts of interest.

## Data Availability

Data sharing not applicable to this article as no datasets were generated or analysed during the current study.
